# FOXA1‐induced LINC00621 promotes lung adenocarcinoma progression via activating the TGF‐β signaling pathway

**DOI:** 10.1111/1759-7714.14986

**Published:** 2023-06-05

**Authors:** Jingjing Wei, Haiyang Yu, Tan Liu, Ziming Wang, Chuandong Lang, Yueyin Pan

**Affiliations:** ^1^ Department of Tumor Chemotherapy, The First Affiliated Hospital of USTC, Division of Life Sciences and Medicine University of Science and Technology of China Hefei China; ^2^ Department of Medical Oncology 901 Hospital of Joint Logistics Support Force of People Liberation Army Hefei China; ^3^ Department of Orthopedics, The First Affiliated Hospital of USTC, Division of Life Sciences and Medicine University of Science and Technology of China Hefei China

**Keywords:** FOXA1, LINC00621, lung adenocarcinoma, MiR‐34a‐5p, TGF‐β

## Abstract

**Background:**

Lung adenocarcinoma (LUAD) is highly malignant and associated with poor prognoses in patients worldwide. There has been widespread recognition that lncRNAs are tightly linked to LUAD tumorigenesis and development. Here, we identified that the LINC00621 level was increased in LUAD tissues and concerned with the poor prognoses in LUAD patients.

**Methods:**

Bioinformatical analysis and RT‐qPCR determined the level of LINC00621 in LUAD tissues and cell lines. The admeasurement of the proliferation, migration, and invasion abilities of LUAD cells was utilized in the CCK8 and Transwell formulas. Luciferase reporter assay was used to corroborate the downstream target genes of LINC00621. The phosphorylated SMAD3 protein was tested by Western blotting assay. The impression of LINC00621 knockdown on LUAD tumor growth and metastasis put into effect by murine models. ChIP‐qPCR assay was carried out to verify the transcriptional regulation by FOXA1 on LINC00621.

**Results:**

In vitro, the knockdown of LINC00621 significantly reduced the proliferative, migrating, and invasive abilities, the same was true for tumorigenesis and metastasis in vivo. MiR‐34a‐5p as a straight target of LINC00621 was ascertained, and LUAD patients with inferior miR‐34a‐5p levels had undesirable prognoses. Furthermore, TGFBR1 is an immediate and functional connection site of miR‐34a‐5p. Collectively, LINC00621 can sponge miR‐34a‐5p and upregulate TGFBR1 levels, which further sensitized TGF‐β signaling pathway. Finally, it was revealed that FOXA1 transcriptionally upregulated LINC00621.

**Conclusion:**

This study uncovered that FOXA1‐induced LINC00621 promotes LUAD progression via the miR‐34a‐5p/TGFBR1/TGF‐β axis, and is one novel therapeutic target that may be used in LUAD treatment.

## Graphical abstract

In conclusion, our study revealed that FOXA1 transcriptionally upregulated LINC00621. And miR‐34a‐5p as a straight target of LINC00621 was ascertained, and LUAD patients with inferior miR‐34a‐5p levels had undesirable prognoses. Furthermore, TGFBR1 is an immediate and functional connection site of miR‐34a‐5p. Collectively, LINC00621 can sponge miR‐34a‐5p and upregulate TGFBR1 levels, which further sensitized TGF‐β signaling pathway. This study uncovers that FOXA1‐induced LINC00621 promotes LUAD progression via miR‐34a‐5p/TGFBR1/TGF‐β axis.

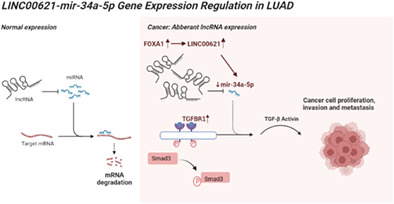

## INTRODUCTION

As lung cancers are generally asymptomatic, most cases are diagnosed at advanced stages, and the surgical therapy used is no longer curative. The five‐year survival rate for lung cancer is only 5%, and it is the leading cancer‐related death in the world. Despite this, about 30%–55% of patients initially diagnosed with early‐stage cancer will develop metastatic disease in the future.^1^, ^2^, ^3^ In terms of histology, non‐small cell lung cancer (NSCLC) contributes 80% or more.^4^, ^5^ The most universal histological subtype of NSCLC is lung adenocarcinoma (LUAD).^6^ Currently, the treatment methods for NSCLC span surgery, chemoradiotherapy, targeted, or combination therapy.^7^ However, some molecular subtypes of NSCLC do not have a specific targeted therapy, and chemotherapy and radiotherapy are ineffective in treating patients with advanced stages of the disease.^8^, ^9^ Therefore, searching for prognostic biomarkers is prominent in effectuating maximum beneficial therapeutic strategies.

Recently, long non‐coding RNAs (LncRNAs) have been found to be potentially pivotal moderators of biological processes.^10^ The lncRNA class is composed of RNA molecules over 200 nucleotides long and without a protein‐coding function.^11^ It has a low level in the body but displays more cell type‐specific expression profile than the protein‐coding genes.^12^, ^13^ Some lncRNAs engage chromatin modifiers in interactions with nearby genes to either boost or repress transcription. Furthermore, some lncRNAs, like the NEAT1 lncRNA, which has been linked to several cancer types, nucleate the formation of subnuclear domains by luring specific proteins to those sites. The resulting domains serve as multifunctional gene regulatory structures that affect transcription and post‐transcriptional regulation. Other lncRNAs are exported to the cytoplasm, where they can engage in interactions with particular proteins to influence signaling pathways, regulate the translation of particular mRNAs, or serve as microRNA sponges. They frequently have an impact on either oncogenes or tumor suppressor genes.^14^, ^15^ Even though lncRNAs work in a variety of ways, more consideration has been given to the competitive endogenous RNA (ceRNA) concept. This hypothesis describes a complex post‐transcriptional regulatory network mediated by microRNAs (miRNAs): protein‐coding mRNAs and lncRNAs compete for binding to miRNAs and thereby functionally liberate mRNA targeted by the miRNAs by sharing one or more miRNA response elements (the miRNA binding sites).^16^ The idea has the obvious implication that lncRNAs should now be thoroughly investigated as potential tumor suppressors or oncogenes through their ceRNA function dependent on the role of mRNAs that they regulate.^17^ In light of mounting evidence the important biological functions of lncRNAs in lung cancer are now implicated. They have preternatural abilities enabling them to cause changes in tumors and exert their regulation through a different mechanism.^18^, ^19^ LINC00621 is the latest lncRNA to be discovered that is abnormally expressed in various malignancies, and LINC00621 expression has been found to be increased in LUAD tissues. Meanwhile, an increase in LINC00621 expression has also been linked with the negative prognosis of patients. However, its molecular mechanism in LUAD has not yet been clarified.

The microRNA molecule (miRNA), which is a noncoding RNA, has a short and highly conserved sequence (22 nucleotides).^20^ It negatively regulates mRNA stability and/or represses mRNA translation, thereby exerting the functions of affecting cell apoptosis, cell proliferation, and differentiation.^21^, ^22^ The irregular expression or dysfunction of miRNAs has erstwhile been traced in numerous cancers and the latent mechanisms of their extensive involvement in cancer.^23^ Multiple explorations have manifested that miRNA plays a vital role in each step of LUAD progression.^24^, ^25^ Furthermore, miR‐34a‐5p is potentially upregulated by p53, a well‐known tumor suppressor. Several lncRNAs can participate in cell processes by targeting miR‐34a‐5p, including LINC00662,^26^ LncRNA 1700020I14Rik,^27^ LINC01106,^28^ LncRNA XIST,^29^ and LINC00665.^30^ In addition, a unique insight to enhance prostate cancer treatment is provided by the lncRNA DANCR/miR‐34a‐5p axis, which has been found to increase docetaxel resistance in prostate cancer by targeting JAG1.^31^ By sponging miR‐34a‐5p, lncRNA TP73‐AS1 promoted NSCLC cell motility, proliferation, and resistance to DDP, although it prevented apoptosis.^32^ By modulating signaling via the lncRNA SNHG7/miR‐34a‐5p pathway, quercetin has been reported to reduce the growth of NSCLC cells and cause them to undergo apoptosis.^33^

The transforming growth factor beta (TGF‐β) family includes TGF‐βs, activins or inhibits bone morphogenetic proteins, and müllerian inhibitory factor, which are closely related to many biological processes.^34^ As a result of the TGF‐β1/SMAD signaling process, TGF‐β1 first activates intracellular signals by integrating with TGFBRII, then activates TGFRI kinase, resulting in SMAD phosphorylation. Then, SMAD activation regulates the expression of hundreds of downstream target genes.^35^, ^36^ Certainly, SMAD‐independent pathways can also be activated besides typical SMAD‐dependent.^37^ During the early stage of tumorigenesis, TGF‐β dresses up as a tumor suppressor, yet a high concentration of TGF‐β is associated with EMT and angiogenesis in the later stage of cancer.^38^, ^39^ The TGF‐β signaling pathway could be regulated and controlled by LINC00621 in our study. The forkhead box A1 (FOXA1) protein as a member of a set of peculiar transcription factors (TF) can initiate chromatin remodeling, which leads to the accessibility of other transcription factors in the region.^40^ Several studies have demonstrated that FOXA1 controls growth and cellular identity in LUAD.^41^ On the basis of this study, we will have a preferable comprehension of the possible mechanism of LINC00621 in regulating LUAD, as well as potential targets for novel therapeutics.

## METHODS

### Cell culture

Human lung cancer cell lines A549 and HCC827 were obtained from ATCC. The aforementioned cells were grown in 1640 medium (Gibco), containing 1% penicillin and streptomycin and 10% fetal bovine serum (FBS) (Gibco). These cells were grown in an incubator.

### Tissue samples

Ten pairs of tumor and matched normal tissue samples, and tumor tissue samples of 14 patients with metastasis and 16 patients without metastasis were collected from the Provincial Hospital Affiliated with the University of Science and Technology of China. The information about the patient samples and clinical data for the tissue is shown in Table 1. Patient samples without metastasis indicate patient samples without lymph node metastasis, which was confirmed by physical and imaging examination, and surgical exploration The ethics committee of the institution and the declaration of Helsinki approved all research plans involving participants in human activities. All patients provided signed informed consent.

**TABLE 1:** Details of patient samples used in this study.

Number	Lymph node metastasis	Gender	Age	T	N	M
P1	No	Male	59	1	0	0
P2	No	Female	56	1	0	0
P3	No	Female	54	1	0	0
P4	No	Female	58	1	0	0
P5	No	Male	60	1	0	0
P6	No	Female	70	3	0	0
P7	No	Male	73	1	0	0
P8	No	Female	73	2	0	0
P9	No	Male	30	4	0	0
P10	No	Female	67	4	0	0
P11	No	Male	73	2	0	0
P12	No	Male	71	2	0	0
P13	No	Male	46	2	0	0
P14	No	Male	60	2	0	0
P15	No	Male	65	3	0	0
P16	No	Male	51	3	0	0
P17	Yes	Female	67	2	1	0
P18	Yes	Female	70	1	2	0
P19	Yes	Male	61	2	1	0
P20	Yes	Female	43	2	2	0
P21	Yes	Female	67	3	1	0
P22	Yes	Male	50	1	2	0
P23	Yes	Female	43	2	1	0
P24	Yes	Male	40	3	2	0
P25	Yes	Female	49	2	1	0
P26	Yes	Female	62	4	2	0
P27	Yes	Female	64	4	1	0
P28	Yes	Male	69	1	1	0
P29	Yes	Female	69	2	1	0
P30	Yes	Female	43	1	1	0

### Quantitative real‐time polymerase chain reaction (qRT‐ PCR)

QRT‐PCR was practiced according to a former study.^42^ Briefly, RNA was isolated using TRIzol reagent (Invitrogen). A reverse transcription kit (Takara) and an SYBR‐Green PCR kit (Takara) based on CFX96 Real‐Time System C1000 Cycler (Bio‐Rad Laboratories) were applied. GAPDH and U6 served as internal controls. To ascertain the amount of miR‐34a‐5p, we used the Bulge‐Loop has‐miR‐34a‐5p Primer Set and Bulge‐Loop miRNA qRT‐PCR starting kit (Ribobio). The primers were obtained from Sangon Biotech (China) and are shown in Table 1.^38^

### Plasmid and transfection

Two shRNAs against LINC00621 were obtained from BOXBIO and the sequences were as follows: shRNA#1, 5′‐ACCGGTGGCTCTCGATGTCCGAGAAGCTT.

CAAGAGAGCTTCTCGGACATCGAGAGCCTTTTTTGAATTC‐3′; shRNA#2, 5′‐ACCGGTAGCTAGTTGTTAATCTTAACTTTCAAGAGAAGTTAAGATTAACAACTAGCTTTTTTTGAATTC‐3′. siRNA for FOXA1 was purchased from RioBio and the target sequence is 5′‐GCGTACTACCAAGGTGTGTAT‐3′. Cell transfection was implemented as previously described.^43^

### Western blotting

Western blotting was conducted with reference to a previous study.^44^ Antibodies for SMAD3, p‐SMAD3, GAPDH, and P84 were purchased from Abcam.

### Cell counting kit‐8 (CCK‐8) assay

A CCK‐8 kit (Biosharp) assay was used to evaluate cell viability and performed as previously described.^45^

### Colony formation

For the colony formation assay, 100 cells were cultivated in six‐well plates and incubated for 2 weeks. After washing with phosphate buffered saline (PBS), 4% paraformaldehyde was added, and the cells were stained with 0.1% crystal violet (1 mg/mL) for 20 min.

### Migration and invasion assays

A transwell assay was performed and the method used complied with that in an earlier investigation.^46^ The pertinent reagent sources were matrigel and 8 mm membrane filter inserts (Corning) (BD Biosciences).

### In vivo model

All mice tests were authorized by the Provincial Hospital Affiliated with the University of Science and Technology of China's Institutional Animal Care and Use Committee (no. 2020‐N(A)‐116). Ten male BALB/c mice (5 weeks old) were randomly split into two groups. A549‐NC and A549‐sh1 cells (2 × 10^6^ cells) were subcutaneously injected into the lower flanks of mice (*n* = 5). Then, every two to 3 days, tumor volumes were calculated using caliper measurements. For the lung metastasis model, 2 × 10^6^ viable cells were injected into the tail veins of mice in both groups (*n* = 5). All animal tests were conducted at the Laboratory Animal Center. Mouse anesthesia was performed using isoflurane gas. Finally, the animals were sacrificed using carbon dioxide.

### Cytoplasmic and nuclear RNA fractionation and RNA FISH

The Ambion PARIS system was used to perform a nuclear cytoplasm separation test, and RT‐PCR was used to extract RNA, with GAPDH and U6 as the respective positive controls. A FISH Kit (RiboBio) was used to perform RNA fluorescence in situ hybridization (RNA FISH).

### Luciferase reporter assay

A luciferase reporter assay was executed based on a previous study.^44^ LINC00621‐wt/mut, TGFBR1‐wt/mut, and p(CAGAC)12‐luc luciferase reporter gene plasmid were used in this study. Using a dual‐luciferase reporter assay kit, both the luciferase and Renilla signals were calculated 36 h after transfection (Promega).

### Chromatin immunoprecipitation‐qPCR (ChIP‐qPCR) assay

A ChIP assay was performed to detect transcription factor binding sites. An EZ ChIP kit (Merck Millipore) was used and this method has been previously reported.^42^ The DNA fragments were immunoprecipitated utilizing IgG (Abcam) or FOXA1 (CST) antibodies. The primers are shown in Table 2.

**TABLE 2:** List of primers used for qPCR.

Primer	Sequence (5′–3′)
LINC00621‐F	CCTGTTGGAAGCAAGCTGAA
LINC00621‐R	ATTGGGTTCCCAGACAACGA
TGFBR1‐F	GACAACGTCAGGTTCTGGCTCA
TGFBR1‐R	CCGCCACTTTCCTCTCCAAACT
GAPDH‐F	GTCTCCTCTGACTTCAACAGCG
GAPDH‐R	ACCACCCTGTTGCTGTAGCCAA
U6‐F	CTCGCTTCGGCAGCACAT
U6‐R	TTTGCGTGTCATCCTTGCG
ChIP‐qPCR	
P1‐F	GTGGACAGCACACGAGGCTT
P1‐R	ACCACCACTTTCCCAGACGC
P2‐F	TGGTCCGGTGACATCATTTTGC
P2‐R	TCTTAAGCTGCACTACAAATGCCT
P3‐F	TGAGGGAAGTTACCAAGGCAGC
P3‐R	CCCCACAGAGGCCTTCAATCC
P4‐F	GTGAGTGTGTGCGTGTGTGC
P4‐R	AATCAGGCCCCCTTCTCCCC

Abbreviation: ChIP‐qPCR, chromatin immunoprecipitation‐quantitative polymerase chain reaction.

### Statistical analysis

GraphPad 8.0 software was used for statistical analysis and the data are shown as mean ± SD. A student's *t*‐test was used to compare the means between two groups, while one‐way ANOVA was applied to stack up multiple groups. *p*‐values < 0.05 were considered statistically significant.

## RESULTS

### LINC00621 expression is increased in LUAD and connected with an inferior prognosis in patients

**FIGURE 1:**
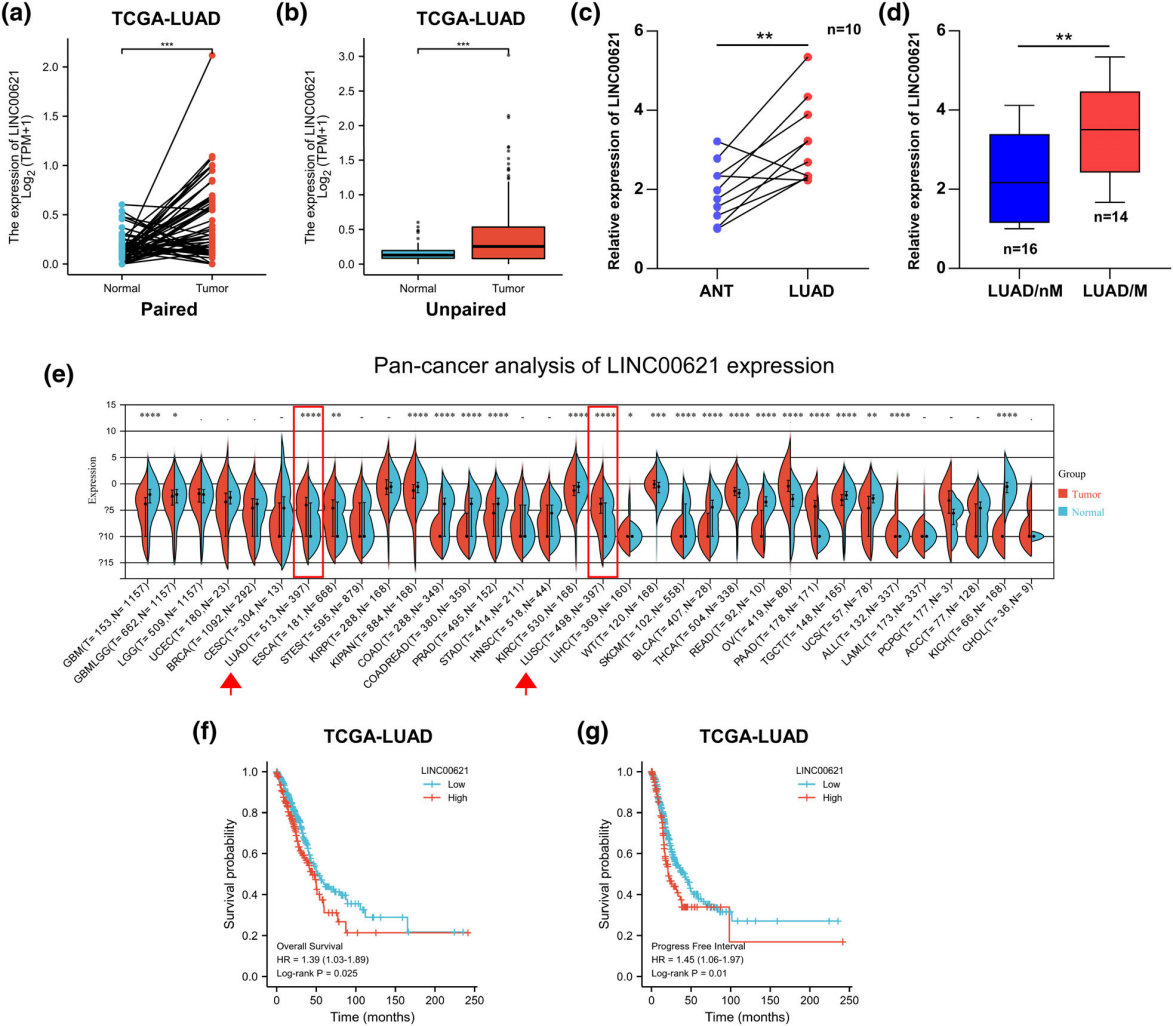
LINC00621 is aberrantly overexpressed in lung adenocarcinoma (LUAD) and is associated with poor prognosis. (a, b) LINC00621 expression in tumor and normal tissues included paired and unpaired from TCGA. (c) The relative LINC00621 expression was analyzed by quantitative reverse transcription‐polymerase chain reaction (qRT‐PCR) in the tumor and adjacent normal tissues (*n* = 10). *(d) The relative LINC00621 expression analyzed in nonmetastatic LUAD patient tissue samples (*n* = 16) and metastatic LUAD patient tissue samples (*n* = 14) by qRT‐PCR. (e) The pan‐cancer analysis of LINC00621 expression. (f, g) Kaplan–Meier analysis showed that elevated expression of LINC00621 was associated with overall survival and progression‐free interval in LUAD patients. Data shown are mean ± SD (*n* = 3). *p < 0.05; **p < 0.01; ***p < 0.001.

Through screening of lncRNAs differentially expressed in LUAD, in the TCGA, we found the LINC00621 levels were remarkably augmented in tumor tissues compared with adjacent normal tissues (Figure 1a,b). Subsequently, the LINC00621 contents were determined in our patient tissues by qRT‐PCR analysis. In contracted adjacent normal tissues, the content of LINC00621 was higher in paired LUAD tissues (Figure 1c). In addition, we traced that high LINC00621 expression was more prevalent in LUAD tissues with metastasis relative to LUAD tissues without metastasis (Figure 1d). Based on the pan‐cancer analysis, we spotted that LINC00621 was upregulated in multiple human cancers, including LUAD and LUSC (Figure 1e). To further confirm the clinical relevance of LINC00621 in LUAD patients, we executed survival analysis stem from TCGA database and identified that LUAD patients with high LINC00621 expression had a worse prognosis of shorter overall survival and progression‐free survival (PFS) (Figure 1f,g). Therefore, these results signify LINC00621 expression is increased and has a bearing on the poor prognosis of LUAD patients.

### LINC00621 facilitates the proliferation of LUAD cells in vitro and in vivo

**FIGURE 2:**
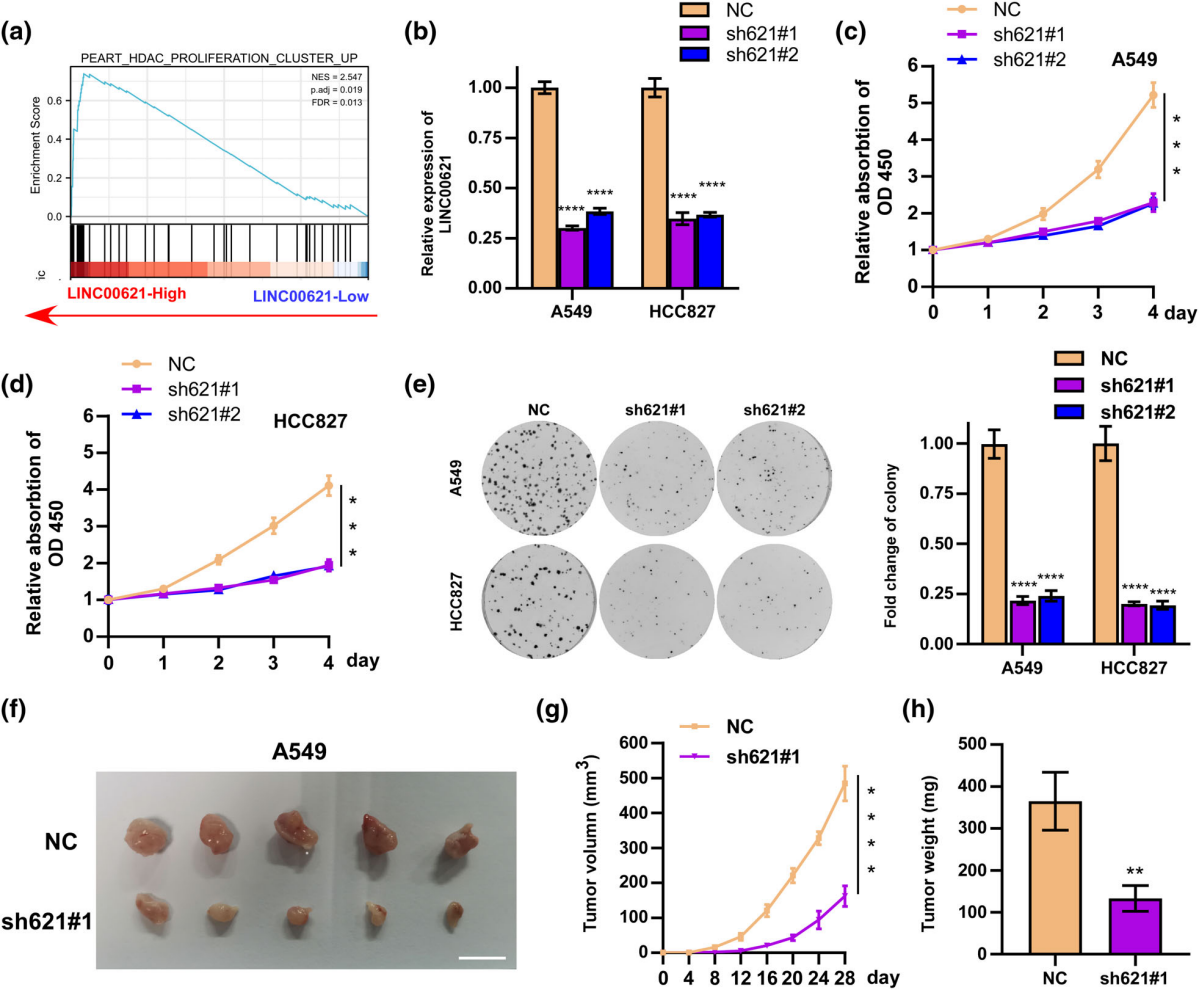
LINC00621 facilitates lung adenocarcinoma (LUAD) cell proliferation in vitro and in vivo. (a) Proliferation enrichment analysis of LINC00621. (b) The expression of LINC00621 was knocked down by two different shRNAs in both HCCC827 and A549 cells. (c, d, e) The proliferation ability of HCC827 and A549 cells after knockdown LINC00621 expression was determined by cell counting kit‐8 (CCK‐8) and colony formation assays. (f) A549 cells treated with sh‐NC lentivirus were inoculated subcutaneously into the left flanks and treated with sh‐1 lentivirus into the right flanks of nude mice (*n* = 5). (g) Tumor volume. (h) Tumor weight. Data shown are mean ± SD (*n* = 3). *p < 0.05; **p < 0.01; ***p < 0.001; ****p < 0.0001, compared with the normal control group.

To probe the function of LINC00621 in LUAD, gene set enrichment analysis (GSEA) found LINC00621 to be positively associated with tumor cell proliferation (Figure 2a). Then, we selected A549 and HCC827 cells in the subsequent experiment. Both cells were transfected with two sh‐LINC00621 plasmids, and the qRT‐PCR results verified the efficiency of LINC00621 knockdown (Figure 2b). Then, CKK‐8 and colony formation assays attested the function of LINC00621 in LUAD cell proliferation. The results proved that low LINC00621 could prohibit the propagation activity of A549 and HCC827 cells (Figure 2c,d), which was also confirmed by colony formation assay (Figure 2e). Subsequently, we structured mouse subcutaneous xenograft models. The shLINC00621‐1 group owned lesser tumor volumes, lower tumor poundage, and lower tumor growth rate (Figure 2f–h). Overall, LINC00621 facilitates the proliferation of LUAD cells.

### LINC00621 knockdown inhibited invasion of LUAD cells in vitro and tumor metastasis in vivo

**FIGURE 3:**
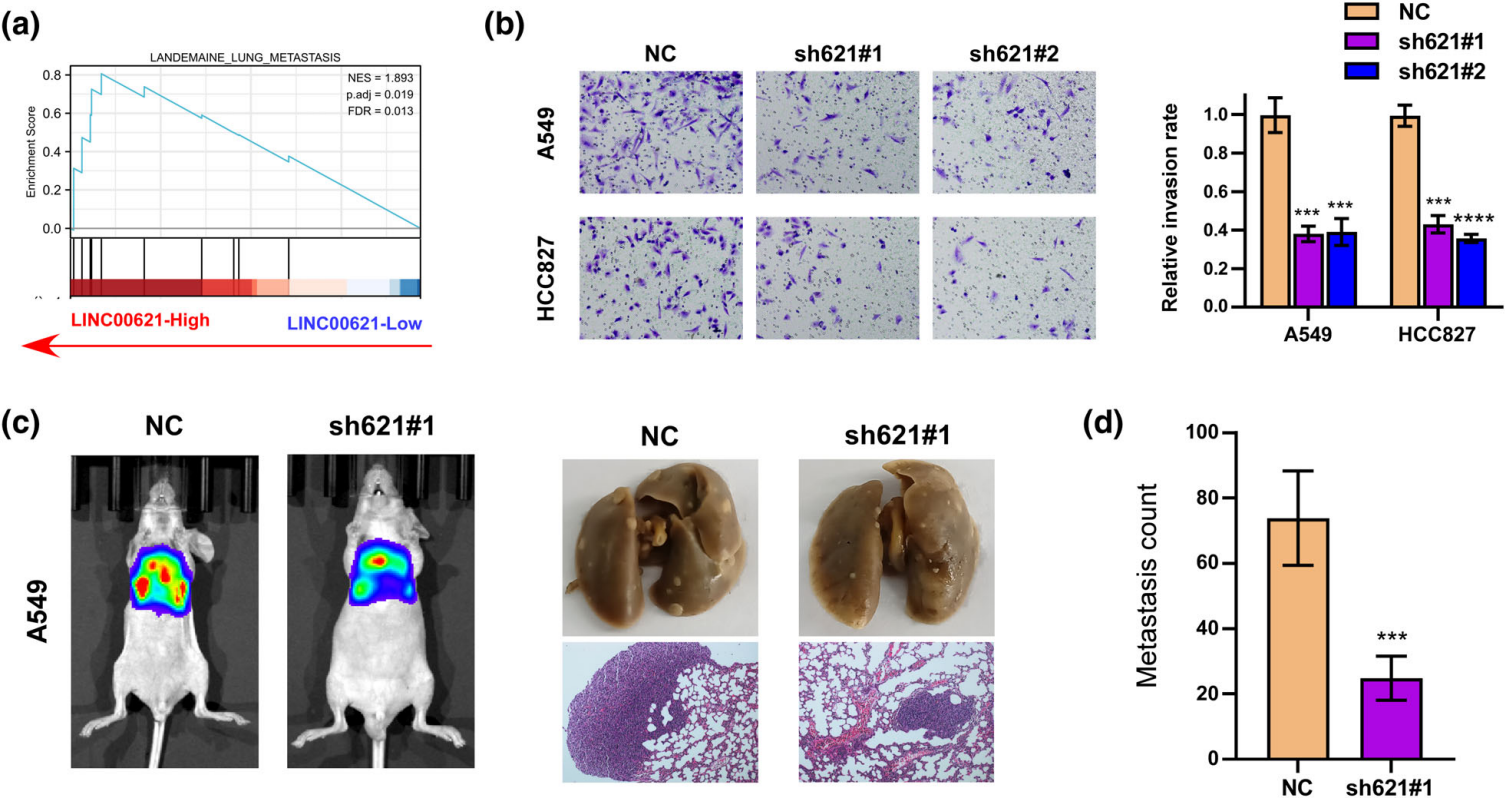
LINC00621 knockdown inhibited the migration and invasion of lung adenocarcinoma (LUAD) cells in vitro and in vivo. (a) Enrichment analysis of LINC00621 metastasis. (b) A transwell assay was used to quantify the migration and invasion of A549 and HCC827 cells with LINC00621 knockdown. (c) Imaging of lung metastasis in mice and lung tissue section. (d) Metastasis count analysis of sh‐1 and NC group. Data shown are mean ± SD (*n* = 3). *p < 0.05; **p < 0.01; ***p < 0.001; ****p < 0.0001.

Meanwhile, the GSEA assay indicated LINC00621 was related to tumor metastasis (Figure 3a). The transwell test interpreted that LINC00621 knockdown inhibited invasion in HCC827 and A549 cells (Figure 3b). Nude mouse lung metastasis models were constructed to further validate the impression of LINC00621 on metastatic potential. After the LINC00621 knockdown, the metastasis ability of LUAD cells was significantly decreased (Figure 3c,d). Taking all the results together, we discovered that LINC00621 elevates the tumor cell invasion and tumor metastasis capacity in LUAD.

### MiR‐34a‐5p is a direct target of LINC00621

**FIGURE 4:**
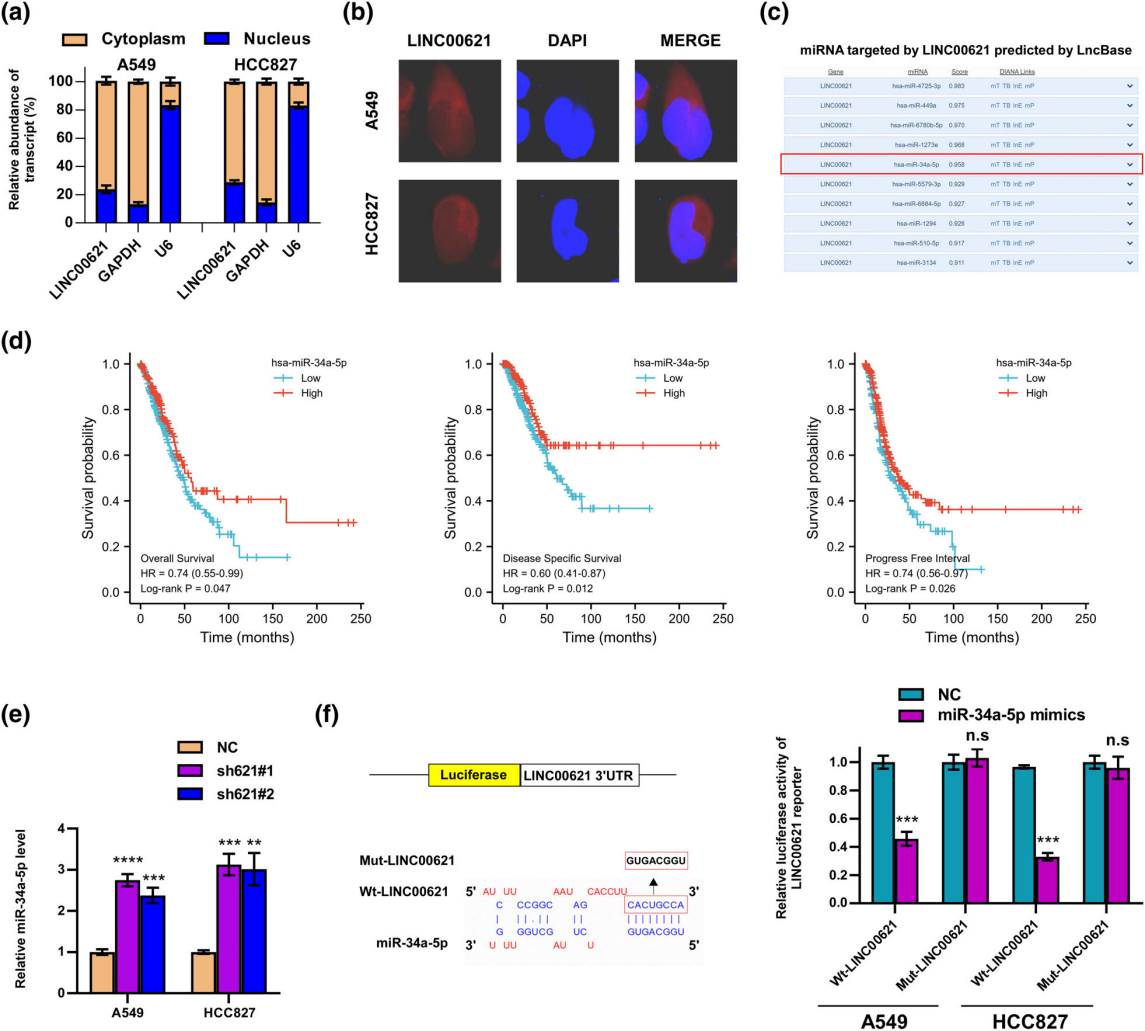
MiR‐34a‐5p was a direct LINC00621 target. (a, b) We analyzed the subcellular localization of LINC00621 in lung adenocaqrcinoma (LUAD) cells using nucleocytoplasmic separation assays and RNA FISH results. (c) MiRNA target by LINC00621 was predicted by LncBase. (d) Survival analysis of miR‐34a‐5p in LUAD. (e) The relative miR‐34a‐5p level in sh‐1, sh‐2 and NC group. (f) Putative target sequence of miR‐34a‐5p on the 3′‐UTR of LINC00621 and detection of luciferase activity by luciferase reporter assay. Data shown are mean ± SD (*n* = 3). *p < 0.05; **p < 0.01; ***p < 0.001; ****p < 0.0001.

First, we analyzed LINC00621's subcellular localization in LUAD cells and the results from nucleocytoplasmic separation and RNA FISH assays indicated that it was predominantly situated in the cytoplasm (Figure 4a,b). Evidence has accumulated that LncRNAs may be portrayed as ceRNAs in modulating the biological functions of miRNAs.^16^ Then, the LncBase database was used to predict the target miRNAs of LINC00621 (Figure 4c). Noteworthy, miR‐34a‐5P, one important tumor suppressor, was the potential target of LINC00621. By performing a prognostic analysis, we discovered that patients with LUAD had a worse prognosis when miR‐34a‐5p was knocked down (Figure 4d). Furthermore, it was found that LINC00621 knockdown dramatically augmented the miR‐34a‐5p contents (Figure 4e). The luciferase reporter experiment was used to confirm that miR‐34a‐5p and LINC00621 directly bind to one another. The experiment demonstrated that treatment with miR‐34a‐5p mimics significantly reduced the luciferase activity of the wild‐type LINC00621 reporter while leaving it intact for the mutant version (Figure 4f). Foregoing results confirmed the direct target of LINC00621 is miR‐34a‐5p.

### LINC00621 activates TGF‐β signaling via sponging miR‐34a‐5p

**FIGURE 5:**
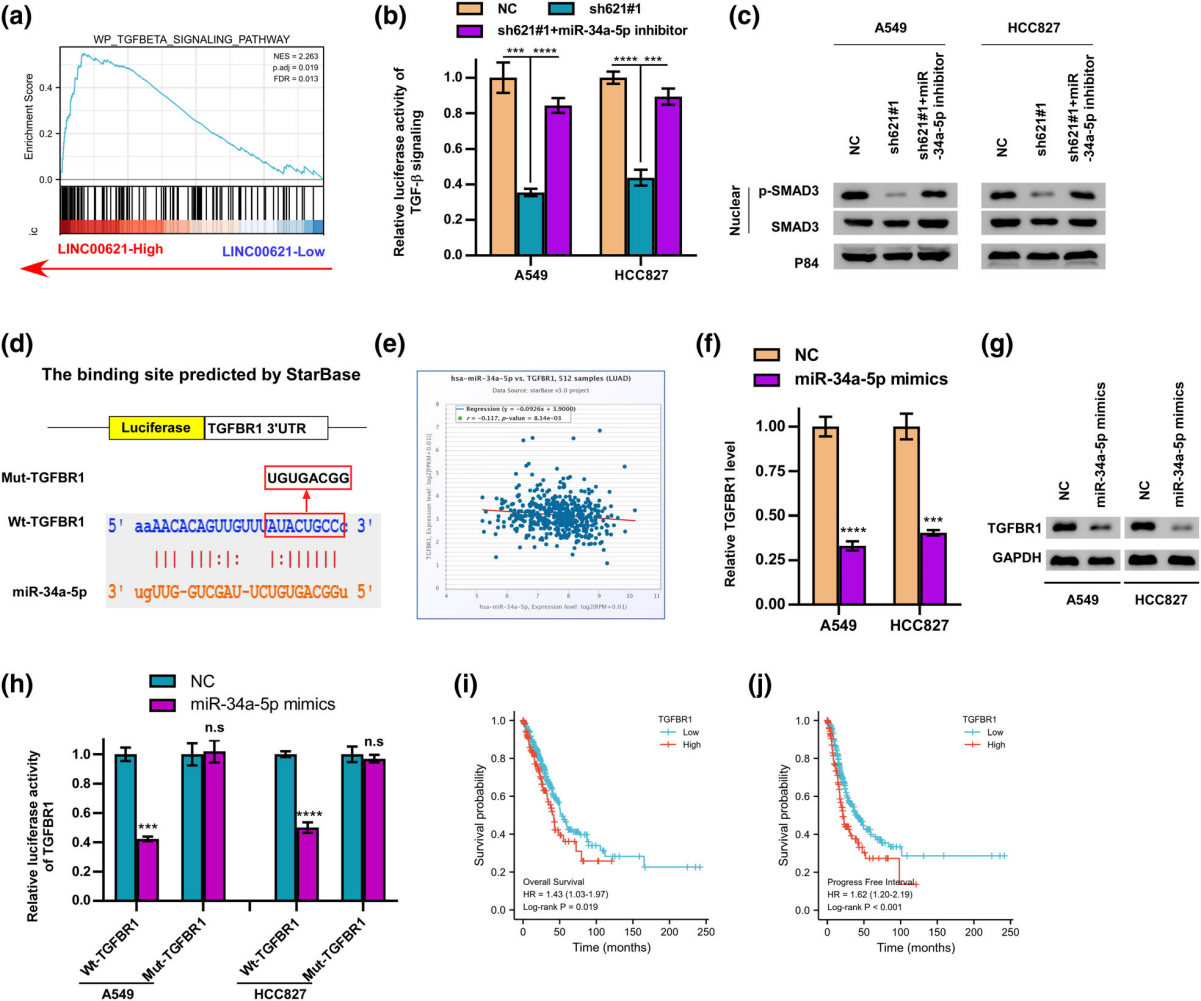
LINC00621 activated TGF‐β signaling via miR‐34a‐5p/TGFBR1. (a) The enrichment of the TGF‐β signaling pathway. (b) TGF‐β signal activity was downregulated by LINC00621 knocked and upregulated by miR‐34a‐5p inhibitor in A549 and HCC827 cells. (c) Western blot experiment showed that protein expression level of p‐SMAD3 repressed by low expression of LINC00621 could be promoted by miR‐34a‐5p inhibitor in A549 and HCC827 cells. There was no change in the level of SMAD protein. P84 protein was used as an internal control. (d) Schematic representation of the predicted binding site between miR‐34a‐5p and TGFBR1 in wild‐ and mutant‐types is shown. (e) Correlation between TGFBR1 and miR‐34a‐5p expression levels. (f, g) TGFBR1 protein and mRNA level of A549 and HCC827 were fortified miR‐34a‐5p mimics. (h) Luciferase reporter activities of wild‐type TGFBR1 and mutant TGFBR1 reporters in A549 and HCC827 cells after adding miR‐34a‐5p mimics. (i, j) Kaplan–Meier analysis showed that elevated expression of TGFBR1 was associated with overall survival and progression‐free interval in lung adenocarcinoma (LUAD). *p < 0.05; **p < 0.01; ***p < 0.001; ****p < 0.0001.

GSEA indicated that high LINC00621 expression sensitizes TGF‐β signaling (Figure 5a). Moreover, the containment of the TGF‐β pathway caused by LINC00621 silencing was reversed following miR‐34a‐5p inhibition (Figure 5b), which was also confirmed by western blotting (Figure 5c). To investigate the mechanism of miR‐34a‐5p regulating TGF‐β signaling and analyze starBase, TGFBR1, one important component of the TGF‐β pathway was screened out to be the potential target of miR‐34a‐5p (Figure 5d). MiR‐34a‐5p expression was negatively correlated with TGFBR1 expression in the TCGA database (Figure 5e). Subsequently, we fortified the miR‐34a‐5p mimics in LUAD cells and detected the diminution of TGFBR1 level (Figure 5f,g). We commenced a luciferase reporter assay to attest that TGFBR1 and miR‐34a‐5p could be combined, and we found that the wild‐type TGFBR1 reporter's luciferase activity was drastically diminished by miR‐34a‐5p, but the control group was unaffected by the treatment of miR‐34a‐5p mimics in A549 and HCC827 cells (Figure 5h). Survival analysis showed TGFBR1 overexpression was interrelated to shorter overall and PFS in LUAD patients (Figure 5 I‐J). Taken together, as miR‐34a‐5p sponges, LINC00621 can activate TGF‐β signaling.

### LINC00621 is transcriptionally activated by FOXA1

**FIGURE 6:**
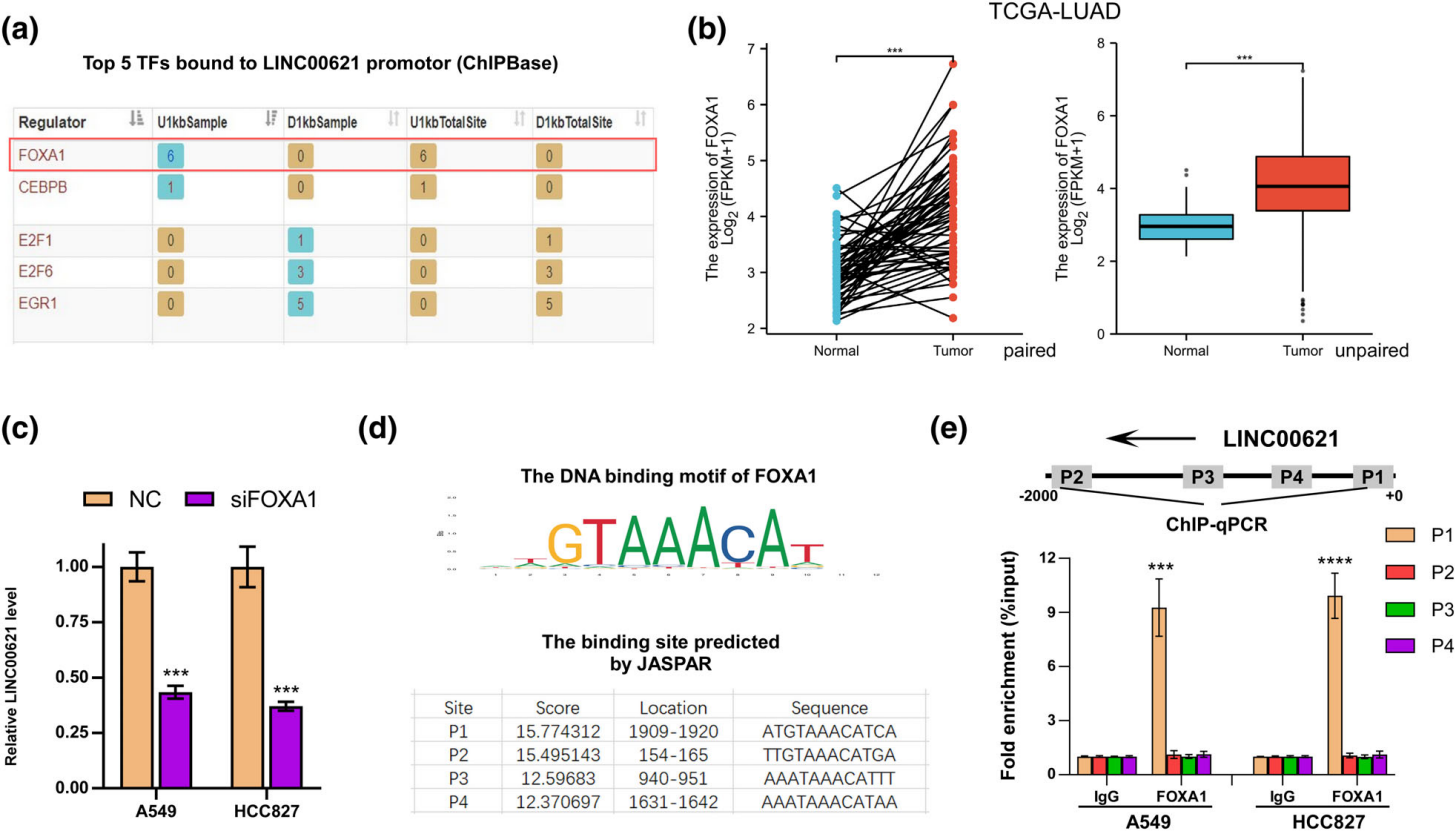
FOXA1 might be the transcription factor of LINC00621. (a) The top five transcription factors bound to LINC00621 promotor are exhibited. (b) The expression of FOXA1 in tumor and normal tissues of lung adenocarcinoma (LUAD) patients in TGGA. (c) The mRNA level of LINC00621 of A549 and HCC827 cells with FOXA1 knockdown. (d) DNA motif of FOXA1 and the binding sites of FOXA1 on the promotor of LINC00621 are shown. (e) ChIP‐qPCR, chromatin immunoprecipitation‐quantitative polymerase chain reaction (ChIP‐qPCR) determined the binding sites of FOXA1. *p < 0.05; **p < 0.01; ***p < 0.001; ****p < 0.0001.

To investigate the mechanism responsible for LINC00621 upregulation, the transcriptional factors bound to the LINC00621 promotor were explored. Based on the prediction of ChIPBase, FOXA1 was the top1 transcriptional factor (Figure 6a). At the same time, compared with LUAD normal tissues, overexpression of FOXA1 in LUAD tumor tissues on the TCGA database (Figure 6b). In LUAD cells, we diminished the FOXA1 level and found FOXA1 depletion caused a low expression of LINC00621 (Figure 6c). The DNA motif of FOXA1 and the binding sites of FOXA1 on LINC00621 promotor were elucidated in Figure 6d. The ChIP‐qPCR assay state clearly that the P1 site was the binding site of FOXA1 on LINC00621 promotor (Figure 6e). These data revealed that FOXA1 transcriptionally upregulates LINC00621 in LUAD.

## DISCUSSION

It is increasingly apparent that dysregulation of lncRNAs promotes multiple types of cancers, including LUAD. Plenty of lncRNAs probably serve as diagnostic and potential detection biomarkers and therapeutic targets in human cancers.^47^ Our study showed a positive correlation between high LINC00621 expression and poor prognosis in LUAD patients. Therefore, in the treatment of LUAD, LINC00621 probably be a potential therapeutic target. Subsequently, we identified LINC00621 as a functional factor in LUAD. After the downregulation of LINC00621, LUAD cells were suppressed in terms of proliferation, invasion, and migration. Therefore, our study uncovered that LINC00621 is a tumor‐promoting factor in LUAD.

According to our localization experiments, LINC00621 is present in the cytoplasm. The ceRNA hypothesis indicates that lncRNAs can participate in miRNA‐mRNA interactions to regulate mRNA expression via miRNA response elements.^16^, ^48^ A miRNA is a form of noncoding RNA that may combine with mRNAs 3UTR, resulting in mRNA degradation or translation inhibition. It plays a meaningful role in various stages of cells.^49^ Extensive studies have indicated that the disorder of miRNA biogenesis enzymes and tumor‐suppressive miRNA (such as miR‐34 family) dysregulation partake in cancer initiation and progression.^50^ It is well known that miRNA is closely related to cancer progression, including apoptosis, EMT, or angiogenesis. It also influences tumor immunity, taking part in immune escape and the immune surveillance process. Obviously, miRNAs may contribute to the development of cancer, but they may also have inhibitory effects, depending on whether the targeted mRNA is an oncogene or a tumor suppressor gene.^51^ Moreover, miRNAs may participate in cancer through a ceRNA manner (also known as miRNA sponge interactions).^52^, ^53^ We found that LINC00621 could bind to miR‐34a‐5p as an endogenous RNA to produce an oncogenic effect. A previous study also confirmed that lncRNA XIST serves as a ceRNA for miR‐34a, finally accelerating thyroid cancer development.^54^

TGF‐β/SMADs signaling pathways play a part in replication, differentiation, and cancer initiation and progression.^55^ We discovered that knockdown of LINC00621 decreased p‐SMAD3 content. Later, there was a direct binding of TGFBR1 and miR‐34a‐5p identified and the decrease of miR‐34a‐5p content caused TGF‐β signaling activation. Our current results show upregulation of LINC00621 can sensitize the TGF‐β signaling. In addition, a previous study has revealed the essential role of LINC00941 in metastatic CRC by activating the TGF‐β/SMAD2/3.^56^ Eventually, we spotted that FOXA1 was effective as the transcription factor for LINC00621. According to previous reports, FOXA1 is an oncogene in various cancers, including prostate cancer^57^ and LUAD.^58^ Meanwhile, FOXA1 induced the transcription of lncRNA MIR99AHG and LINC01207 and played a tumor facilitator in pancreatic cancer and head and neck squamous cell carcinoma.^59^, ^60^

In conclusion, our study reveals one novel mechanism of how LINC00621 promotes LUAD progression via the miR‐34a‐5p/TGFBR1/TGF‐β axis, which suggests LINC00621 may act as one therapeutic target against LUAD.

## AUTHOR CONTRIBUTIONS

JW and HY were responsible for bioinformatic analysis, experimental operation, writing of the study and its subsequent revision. TL and ZW were responsible for tissue specimen collection and qRT‐PCR experiments. YP and CL were responsible for scientific research design, project administration, funding acquisition, data curation, writing‐review and editing.

## FUNDING INFORMATION

Anhui Province Cancer Bioimmunotherapy Clinical Medical Research Center (grant no. 202101B10202005) and The Construction Project of Provincial Key Medical and Health Specialties (grant no. 2021sjlczdzk) supported our study.

## CONFLICT OF INTEREST STATEMENT

There are no material financial or non‐financial interests to disclose for the authors.

## Data Availability

The analyzed datasets created during the study can be obtained from the corresponding author upon reasonable request.
